# Reduced Muscular Carnosine in Proximal Myotonic Myopathy—A Pilot ^1^H‐MRS Study

**DOI:** 10.1002/acn3.70263

**Published:** 2025-11-25

**Authors:** Alexander Gussew, Maryam Kargaran, Maik Rothe, Andreas Deistung, Dietrich Stoevesandt, Walter A. Wohlgemuth, David Strube, Thomas Kendzierski, Anna Katharina Kölsch, Maurits Gerhard Abraham Heuschen, Markus Otto, Alexander Mensch

**Affiliations:** ^1^ University Clinic and Outpatient Clinic for Radiology University Hospital Halle (Saale) Halle (Saale) Germany; ^2^ Halle MR Imaging Core Facility University Medicine Halle (Saale) Halle (Saale) Germany; ^3^ University Clinic and Outpatient Clinic for Neurology University Medicine Halle (Saale) Halle (Saale) Germany; ^4^ Heimer Institute for Muscle Research Bochum Germany

**Keywords:** 1H‐MR spectroscopy, carnosine, muscle, myotonic dystrophy type 2, PROMM

## Abstract

**Objective:**

Myotonic dystrophy type 2 (proximal myotonic myopathy, PROMM) is a progressive multisystem disorder with muscular symptoms (proximal weakness, pain, myotonia) and systemic manifestations such as diabetes mellitus, cataracts, and cardiac arrhythmias. A hallmark feature is the selective degeneration of type‐II fibers, likely driven by chronic myotonia and sustained metabolic stress. In this study, proton magnetic resonance spectroscopy (^1^H‐MRS) was applied to assess intramuscular carnosine as a potential noninvasive marker of type‐II fiber integrity, alongside extramyocellular lipids (EMCL) and intracellular pH. We hypothesized that carnosine would be reduced in PROMM as a consequence of type‐II fiber loss.

**Methods:**

Seventy participants (27 genetically confirmed PROMM patients, 43 healthy volunteers) underwent localized ^1^H‐MRS of the quadriceps muscle at 3 T using a short‐TE PRESS sequence. To ensure reliable carnosine quantification, spectra with voxel fat fraction ≥ 40% were excluded, yielding a final cohort of 19 patients and 19 matched healthy volunteers. Metabolites were quantified relative to total creatine, and exploratory correlations were assessed.

**Results:**

PROMM patients showed significantly reduced carnosine compared with healthy volunteers (−50%, 0.05 ± 0.03 vs. 0.10 ± 0.05; *p* = 0.0022) and markedly elevated EMCL (threefold, 150.6 ± 80.5 vs. 48.6 ± 38.4; *p* = 0.0007). Intracellular pH did not differ between groups. Exploratory analysis revealed a negative correlation between carnosine and EMCL (*r* = −0.50, *p* = 0.03).

**Interpretation:**

This pilot study demonstrates that ^1^H‐MRS can detect reduced intramuscular carnosine in PROMM, consistent with selective type‐II‐fiber loss. Carnosine thus emerges as a potential in vivo biomarker of fiber‐type‐specific degeneration. Validation in larger, longitudinal cohorts is warranted to establish its clinical and translational relevance.

## 1 Introduction

Myotonic dystrophy type 2 (proximal myotonic myopathy, PROMM) is a progressive multisystem disorder caused by a (CCTG)n repeat expansion in the CNBP gene (Cellular Nucleic Acid Binding Protein), characterized by muscular symptoms (myotonia, weakness, and pain in proximal muscles) and systemic manifestations including diabetes mellitus, early‐onset cataracts, and cardiac conduction defects or arrhythmias [1, 2]. Among these symptoms, persistent myotonia represents one of the hallmark features in PROMM [2]. This sustained hypertonic state imposes a chronic metabolic burden particularly on fast‐twitch (type‐II) fibers, which are essential for mobility and postural control. As type‐II fibers rely primarily on anaerobic glycolysis, they are more prone to accumulating metabolic byproducts such as hydrogen ions and lactate during sustained activation [3]. The resulting local acidosis impairs muscle contractile function, disrupts cellular homeostasis, and may ultimately contribute to fiber‐type‐specific degeneration [4]. Indeed, selective loss of type‐II fibers has been observed in PROMM muscle biopsies, alongside increased fat infiltration and fiber‐type switching [5].

Genetic testing permits a definitive diagnosis of the disease, and quantitative fat‐water MR imaging combined with functional assessments enables monitoring of disease progression [6]. However, current approaches to assess muscle integrity—particularly with respect to fiber‐type‐specific pathology—remain limited. Although muscle biopsies provide detailed histological characterization, their invasiveness precludes routine application for longitudinal monitoring.

In this context, proton magnetic resonance spectroscopy (^1^H‐MRS) emerges as a promising tool for the noninvasive assessment of muscle alterations in PROMM. ^1^H‐MRS allows inherent differentiation between intramyocellular (IMCL) and extramyocellular (EMCL) lipid compartments in skeletal muscles, reflecting metabolically active versus structurally infiltrated fat, respectively [7, 8, 9]. Moreover, ^1^H‐MRS enables the quantification of carnosine [10, 11], a histidine‐containing dipeptide that is highly concentrated in type‐II muscle fibers due to its roles as an effective intracellular buffer and antioxidant [12]. Previous studies have applied ^1^H‐MRS to examine carnosine alterations in skeletal muscle under various physiological and pathological conditions, including physical training, dietary interventions, beta‐alanine supplementation, aging, and metabolic diseases such as diabetes [13, 14, 15, 16, 17, 18]. Given the selective vulnerability of type‐II fibers in PROMM, a reduction in intramuscular carnosine may reflect ongoing fiber‐type‐specific degeneration. In addition, the chemical shift of the carnosine HC^2^ peak near 8.0 ppm enables estimation of intracellular pH, providing an indirect measure of local acidosis and metabolic homeostasis in chronically stressed muscle tissue [10].

Against this background, the aim of the present study was to apply localized ^1^H‐MRS to quantify carnosine, intracellular pH, and EMCL in the thigh muscles of PROMM patients and matched healthy volunteers. Based on the presumed selective vulnerability of type‐II fibers in PROMM, we hypothesized lower carnosine concentrations in patients. In addition, we expected elevated EMCL levels as a spectroscopic correlate of structural fat infiltration. Furthermore, assuming altered acidosis of chronically stressed type‐II fibers, we anticipated pH differences between groups. As part of an exploratory analysis, we also examined associations between carnosine and these markers to investigate potential interdependent metabolic alterations.

## 2 Material and Methods

### 2.1 Participants and Clinical Assessment

In this study, a total of 70 subjects were included, 27 PROMM patients (16 female/11 male, median age [Q_1_–Q_3_]: 59.7 [52.9–64.5] years, BMI: 25.4 [22.5–30.2] kg/m^2^) and 43 healthy volunteers (22 female/21 male, 50.1 [38.2–59.1] years, BMI: 24.5 [22.2–26.9] kg/m^2^).

Patient selection was based on routine clinical admissions at the Neuromuscular Centre Halle of the University Clinic and Outpatient Clinic for Neurology, University Medicine Halle (Saale). Inclusion required genetically confirmed CNBP repeat expansion. In addition, patients had to present with clinical features consistent with PROMM (e.g., myotonia, proximal muscle weakness, and/or muscle pain) and electrophysiological evidence of myotonic discharges on needle electromyography. Healthy volunteers were recruited from the institutional staff pool. All healthy volunteers underwent screening to exclude any evidence of neuromuscular disease. All participants were required to meet standard MRI eligibility criteria; individuals with contraindications such as ferromagnetic implants, pacemakers, or pronounced claustrophobia were excluded from the study.

The study was approved by the local ethics committee (protocol No.: 2022‐012) and conducted in accordance with the Declaration of Helsinki. Written informed consent was obtained from all participants.

### 2.2 MR Data Acquisition

**FIGURE 1:**
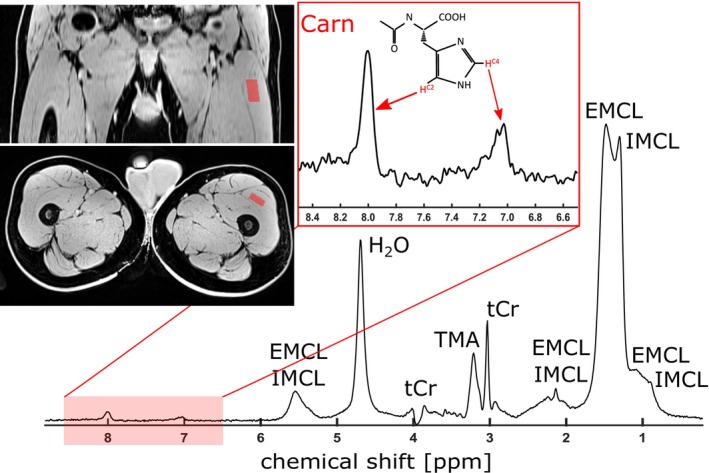
Selected voxel in left quadriceps muscle (red box in anatomical images) and representative ^1^H‐MR spectrum of a healthy volunteer with resonances of carnosine (imidazole singlets Carn^HC4^ at ~7.0 ppm, Carn^HC2^ at ~8.0 ppm), total creatine (at 3 ppm), intra‐ and extracellular lipids (IMCL and EMCL), and trimethyl ammonium compounds (TMA at 3.2 ppm).

^1^H‐MR spectra (echo/repetition times (TE/TR): 30/3000 ms, 84 averages, 2048 signal samples, receiver bandwidth: 2000 Hz, manual B_0_ shimming, water suppression) were acquired from the left quadriceps muscle using a single‐voxel, point resolved spectroscopy (PRESS) localization approach (voxel size: 8.3 mL, see Figure 1). These measurements were part of a multimodal whole‐body MR imaging protocol (~1 h total duration) designed to assess muscular involvement in neuromuscular disorders. Examinations were performed on a 3 T clinical scanner (MAGNETOM Vida, Siemens Healthineers, Erlangen, Germany) using a dedicated combination of receive coils. A 72‐channel spine coil and 36‐channel peripheral angiography coil were used for MRS acquisition in the thigh region. MR spectra were acquired (~10 min) at the end of the protocol to allow sufficient muscle recovery and minimize potential effects of prior activity on metabolic parameters.

Voxel positioning was guided by quantitative water‐fat MR imaging using a 3D multi‐echo Dixon technique (TR: 9.4 ms; TE range: 1.37–7.92 ms, ΔTE: 1.31 ms, flip angle: 4°, voxel size 1 × 1 × 3 mm^3^). Utilizing the resulting water and fat fraction (FF) images, the MRS voxel was placed in a visually homogeneous, fat‐reduced region of the quadriceps muscle to avoid contamination from adjacent tissues.

### 2.3 MR Spectra Analysis

Single‐scan ^1^H‐MRS data were corrected for frequency and phase shifts using in‐house MATLAB scripts (MathWorks Inc., Natick, MA, USA) and then averaged to generate a mean spectrum (see Figure 1). The mean spectra were quantified using the AMARES fitting algorithm (Advanced Method for Accurate, Robust, and Efficient Spectral fitting [19]) implemented in the jMRUI software package (http://www.jmrui.eu/). Among others, the quantified components included imidazole singlets of carnosine (Carn^HC4^ at ~7.0 ppm, Carn^HC2^ at ~8.0 ppm), total creatine (tCr at 3 ppm), and distinct resonances of EMCL (singlets at 1.1, 1.4, 2.3 and 5.5 ppm). The individual EMCL resonances were summed to yield total EMCL (EMCL^sum^) intensities. To account for interindividual variability in absolute signal intensities, metabolite‐to‐creatine ratios were calculated for all participants.

Additionally, the chemical shift (δ in ppm) of the carnosine HC^2^ peak was used to estimate the intracellular pH according to Pan et al. [10]:

(1) $$\mathrm{pH}=6.73+\log_{10}\left(\frac{8.57-\delta }{\delta -7.67}\right)$$

To assess the structural composition within the localized spectroscopy volume, the MRS voxel position was co‐registered to the corresponding multi‐echo Dixon MR images. From the resulting voxel mask, mean intra‐voxel FFs were calculated from the quantitative FF maps.

### 2.4 Study Cohort Selection and Statistical Analysis

Based on predefined anatomical criteria, only PROMM datasets with a mean FF below 40% within the MRS voxel were included in the final analysis. This threshold was applied to ensure reliable quantification of the carnosine signal, which becomes unmeasurable at higher levels of fat infiltration. A healthy volunteer sub cohort was subsequently defined by age‐ and sex‐matching to the selected PROMM patient group.

Group comparisons of Carn^HC2^/tCr and EMCL^sum^/tCr ratios as well as pH values were conducted using paired *t*‐tests (PROMM patients vs. paired healthy volunteers). In addition to *p*‐values, Cohen's *d* was calculated to estimate effect sizes, with values of 0.2, 0.5, and 0.8 representing small, medium, and large effects, respectively [20]. To account for multiple comparisons, *p*‐values were Bonferroni‐corrected, resulting in a corrected significance threshold of *α* = 0.05/3 ≈0.017.

Exploratory correlation analyses were performed to examine the relationships between carnosine levels, EMCL, and pH values in both groups. Pearson's correlation coefficient (*r*) was calculated for each variable pair. Corresponding *p*‐values were adjusted for multiple comparisons using false discovery rate (FDR) correction.

## 3 Results

### 3.1 Sample Size Definition

**FIGURE 2:**
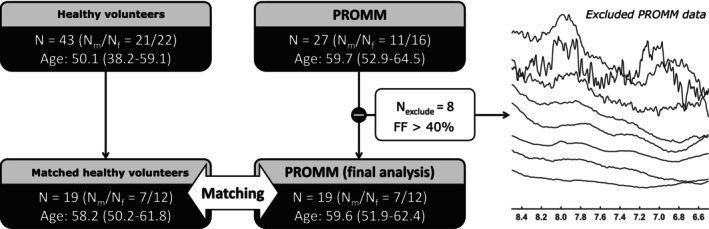
Flowchart illustrating the selection process for PROMM patient datasets based on mean fat content within the MRS voxel. Of the 27 PROMM patients initially enrolled, eight were excluded due to a voxel fat fraction exceeding 40%. The remaining 19 patients were age‐ and sex‐matched with healthy volunteers to form the final study sample. The right‐hand subplot displays the series of excluded PROMM spectra within the carnosine resonance range.

After applying the pre‐specified anatomical criteria, the final study sample comprised 19 PROMM patients and 19 age‐ and sex‐matched healthy individuals. In the initial cohort, the intra‐voxel FF was 0.19 [0.10, 0.34] in PROMM patients (*n* = 27) and 0.05 [0.04, 0.07] in healthy volunteers (*n* = 43), expressed as median [first quartile, third quartile]. Following the exclusion of high‐fat spectra (FF ≥ 40%), the final cohort exhibited lower median FF values in PROMM patients (0.15 [0.09, 0.27]) and slightly higher values in healthy volunteers (0.06 [0.04, 0.08]). The selection process and excluded high‐fat spectra are illustrated in Figure 2.

Spectral quality metrics of the included and excluded datasets confirmed the overall robustness of the measurements. In the final study cohort, the mean ± SD signal‐to‐noise ratios (SNR) were 36.0 ± 21.4 for total creatine (tCr at 3 ppm) and 1.3 ± 0.8 for carnosine (Carn^HC2^ at ~8.0 ppm) in PROMM patients, and 51.5 ± 35.4 and 2.5 ± 1.4, respectively, in healthy volunteers. The corresponding full width at half maximum (FWHM) of the tCr peak was 14.6 ± 2.9 Hz in patients and 14.2 ± 2.1 Hz in controls. In PROMM patient spectra excluded due to high intra‐voxel FFs, the tCr SNR was markedly lower (17.1 ± 9.4) and the FWHM broader (20.6 ± 3.3 Hz), confirming reduced spectral quality in fat‐infiltrated voxels. The SNR was calculated as the ratio between the baseline‐corrected peak magnitude and twice the standard deviation of the noise, determined in a signal‐free spectral region between −3 and −1 ppm.

### 3.2 Group Comparisons

**FIGURE 3:**
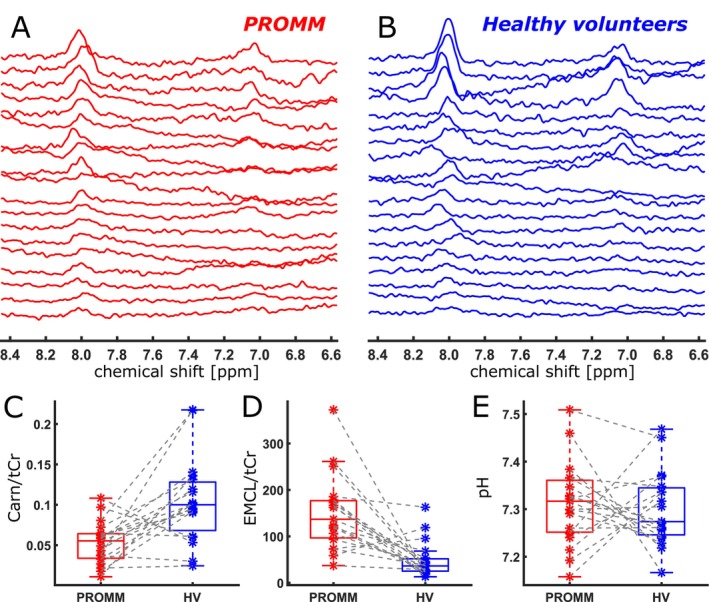
Series of individual ^1^H‐MR spectra from all included PROMM patients (A) and matched healthy volunteers (HV) (B) in the chemical shift range of 6.5–8.5 ppm, illustrating the overall reduction of the carnosine HC^2^ peak (~8.0 ppm) in the PROMM group. (C–E) Boxplots showing distributions of Carn^HC2^/tCr (C), EMCL^sum^/tCr (D), and pH values (E) in PROMM patients (red) and matched healthy volunteers (blue). Individual parameter values for each subject are depicted as asterisks, with gray dashed lines connecting values of the patient—healthy volunteer pair.

Qualitative inspection of the averaged ^1^H‐MR spectra revealed a visible reduction in the carnosine HC^2^ resonance in the PROMM group compared with healthy volunteers (Figure 3A,B). Quantitative analysis further demonstrated significantly lower Carn^HC2^/tCr ratios and significantly higher EMCL^sum^/tCr ratios in PROMM patients, whereas no significant differences in pH values were observed between groups (Figure 3C–E). Consistent with the EMCL findings, FF values in MRS voxels were also significantly higher in PROMM patients compared with healthy volunteers. The assumption of normality of paired differences was verified using the Shapiro–Wilk test, which was not significant for any parameter. Mean values, standard deviations, effect sizes (Cohen's *d* with 95% confidence intervals), and corresponding *p*‐values from paired *t*‐tests are summarized in Table 1.

**TABLE 1:** Group comparisons of Carn^HC2^/tCr, EMCL^sum^/tCr, FF values and pH values in MRS voxel and pH values between PROMM patients and matched healthy volunteers (mean ± standard deviation).

	PROMM (Mean ± SD)	Healthy volunteers (Mean ± SD)	Cohen's *d* (95% CI)	*p*
Carn^HC2^/tCr	0.05 ± 0.03	0.10 ± 0.05	−0.91 (−0.08, −0.02)	0.0033
EMCL^sum^/tCr	150.6 ± 80.5	48.6 ± 38.4	1.08 (55.2, 148.8)	0.0009
FF	0.17 ± 0.11	0.07 ± 0.04	0.84 (0.042, 0.16)	0.0043
pH	7.3 ± 0.09	7.3 ± 0.08	0.09 (−0.05, 0.07)	0.7080

*Note*: Cohen's *d* values indicate effect sizes with 95% confidence intervals. *p*‐values were derived from paired *t*‐tests (Bonferroni‐corrected significance threshold: *α* = 0.017).

### 3.3 Correlation Analysis

**FIGURE 4:**
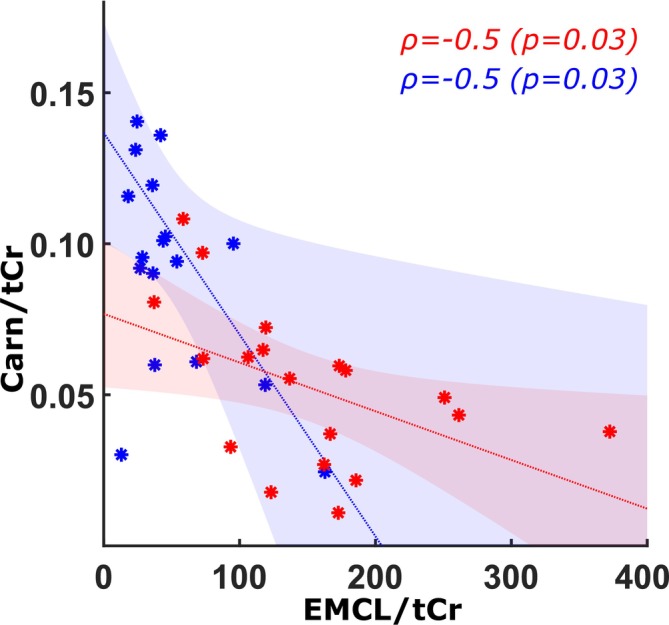
Scatter plots showing the relationships between Carn^HC2^/tCr and EMCL^sum^/tCr in PROMM patients (red asterisks) and healthy volunteers (blue asterisks). For each group, linear regression lines (solid lines) and their corresponding 95% confidence intervals (shaded areas) are shown in matching colors (red for patients, blue for healthy volunteers). 95% confidence intervals were derived using bootstrap resampling (10,000 iterations).

Exploratory analyses revealed a significant negative correlation between EMCL^sum^/tCr and Carn^HC2^/tCr in both PROMM patients and healthy volunteers (both *r* = −0.50, FDR‐corrected *p* = 0.03; Figure 4). Moreover, EMCL^sum^/tCr was positively correlated with FF in PROMM (*r* = 0.5710, *p* = 0.0107) and in healthy controls (*r* = 0.7095, *p* = 0.0007), indicating that higher EMCL levels were consistently associated with greater intramuscular fat infiltration. For all other parameter pairs (Carn^HC2^/tCr vs. pH, EMCL^sum^/tCr vs. pH), no significant correlations were observed after FDR correction.

## 4 Discussion

This study provides the first in vivo evidence that localized ^1^H‐MRS can be used to assess intramuscular carnosine alterations in PROMM. We investigated carnosine, extramyocellular lipids, and intracellular pH in proximal thigh muscles of genetically confirmed patients and matched controls. The quadriceps, a muscle typically affected in PROMM and characterized by a high proportion of type‐II‐fibers [21], was chosen as the target region. To ensure reliable quantification of the carnosine signal, only spectra from voxels with a FF below 40% were considered, resulting in a cohort of 19 PROMM patients and 19 age‐ and sex‐matched healthy volunteers. This strict selection procedure reduced the initial sample size but was necessary to allow accurate carnosine quantification.

In line with our initial hypothesis, PROMM patients exhibited significantly lower Carn^HC2^/tCr ratios compared with healthy volunteers, consistent with the presumed vulnerability of type‐II fibers in this disease. Furthermore, EMCL^sum^/tCr ratios were markedly elevated in PROMM, indicating structural fat infiltration of skeletal muscle. This finding was corroborated by significantly higher FF values in PROMM, and the positive association between EMCL^sum^/tCr ratios and FF values suggests that intramuscular fat accumulation is consistently captured by both spectroscopy and quantitative MRI measures. However, intracellular pH did not differ significantly between groups. Beyond group comparisons, exploratory analyses revealed a robust negative correlation between carnosine and EMCL levels in both patients and healthy volunteers, pointing to a link between fat infiltration in the muscle and fiber‐type‐specific degeneration.

### 4.1 Reduced Muscle Carnosine in PROMM and Potential Confounding Factors

Previous histochemical analyses have shown that type‐II fibers contain approximately twice as much carnosine as type‐I‐fibers [22]. Given this preferential storage, the observed reduction in carnosine in our study presumably suggests a selective loss of type‐II fiber integrity in PROMM. This interpretation aligns with extensive histopathological evidence demonstrating type‐II fiber atrophy as one of the prominent features of PROMM, distinguishing it from other myotonic dystrophies. Numerous biopsy studies, summarized in review articles by Meola [2] and Rimoldi et al. [23], have consistently highlighted selective involvement of type‐II fibers—including atrophy, fiber‐type switching, and increased fat infiltration.

In our study, particular care was taken to match PROMM patients and healthy volunteers by age and sex in order to minimize physiological confounders. This approach is supported by a large ^1^H‐MRS study by Baguet et al. [13], which demonstrated marked sex‐specific trajectories of muscle carnosine across the lifespan. While carnosine levels were comparable in girls and boys before puberty, they rise substantially in males thereafter and remain higher than in females throughout life. Carnosine also declined from adolescence into mid‐adulthood, with the steepest reductions in type‐II‐rich muscles such as the gastrocnemius medialis, consistent with preferential age‐related loss of type‐II fibers. Against this background, the reductions observed in our PROMM cohort substantially exceed the expected age‐related decline (estimated at ~1%–2% per year [13]) and point to disease‐specific mechanisms of type‐II fiber degeneration. In fact, PROMM patients showed approximately 50% lower carnosine levels compared with matched healthy volunteers (Table 1), far beyond what can be explained by normal aging.

Dietary habits may also influence intramuscular carnosine levels. Everaert et al. reported that long‐term vegetarianism (≥ 8 years) is associated with approximately 20% lower muscle carnosine levels compared with those of omnivores [14]. Unfortunately, dietary habits were not assessed in our study, and no matching between patients and healthy volunteers was performed with respect to nutrition. Thus, the presence of vegetarians in either group could represent a residual source of bias. However, the magnitude of the carnosine reduction observed in PROMM patients (~50% compared with healthy volunteers; Table 1) clearly exceeds the dietary effect described by Everaert et al. [14], making it unlikely that differences in nutrition alone can account for our findings.

Other factors known to substantially modulate muscle carnosine include physical training status and oral β‐alanine supplementation [16, 17]. However, in our study, participants neither engaged in competitive sports nor reported β‐alanine supplementation. Therefore, these factors can be considered negligible for the interpretation of our findings.

### 4.2 Elevated EMCL Content in PROMM

The EMCL signal originates from adipocytes located in the interstitial and perimysial spaces of skeletal muscle [8]. Unlike IMCL, which reflects metabolic substrate storage [9], EMCL primarily represents structural fat infiltration and corresponds to the FF measurable with Dixon‐based MRI [24]. In our study, PROMM patients exhibited significantly higher EMCL levels compared with healthy volunteers, consistent with expected progressive fatty infiltration accompanying myofiber degeneration. This aligns with previous Dixon‐MRI studies; in particular, Madrid et al. [25] reported approximately threefold higher FF in both thigh and calf muscles of PROMM patients compared with healthy volunteers—a magnitude consistent with the elevated EMCL levels identified in our study (Table 1).

We also observed a negative correlation between EMCL and carnosine levels across the entire cohort, encompassing both PROMM patients and healthy volunteers (see Figure 4). This suggests a general physiological continuum, whereby loss of type‐II fibers is associated with reduced carnosine and increased fat infiltration. In PROMM, however, this relationship appears to be pathologically accentuated, with markedly reduced carnosine concentrations and elevated EMCL shifting the balance toward advanced muscle degeneration. While a linear model was applied here for simplicity, the relationship between carnosine and EMCL may be influenced by additional factors such as age and sex, and potentially follow a more complex pattern; future studies with larger cohorts will be required to enable multivariable or nonlinear modeling of this association. Therefore, this interpretation should be considered tentative, as it is based on an exploratory observation in low‐size samples and therefore requires systematic validation in future studies.

### 4.3 Intermuscular pH in PROMM

We examined whether persistent myotonia in PROMM is associated with detectable alterations of intramuscular pH. Previous ^1^H‐MRS studies have demonstrated that the chemical shift of the carnosine HC^2^ resonance provides a sensitive marker of fiber type‐dependent pH changes under metabolic stress. Pan et al. [10] first established the validity of this approach, reporting a pH decline from ~7.0 to ~6.1 after exhaustive exercise. Damon et al. [26] further demonstrated that muscle activation can induce a splitting of the HC^2^ resonance, reflecting differential acidification of glycolytic versus oxidative fibers. Just Kukurova et al. [27] confirmed this phenomenon in humans at 7 T, observing significant pH decreases and partial HC^2^ peak splitting in the m. gastrocnemius, a muscle with a relatively high proportion of type‐II‐fibers [21], after prolonged running. In contrast, no splitting was seen in the soleus muscle, consistent with its predominance of type‐I fibers.

Notably, none of our PROMM spectra (see Figure 3) displayed such peak splitting, qualitatively indicating the absence of detectable fiber‐type‐specific pH shifts. Two explanations may account for this. First, our measurements were obtained at rest, where dynamic acid–base alterations are unlikely to manifest, even in the presence of chronic myotonia. Second, as emphasized in previous work, the splitting of the carnosine HC^2^ resonance originates primarily from acidification of type‐II‐fibers [26, 27]. Given the preferential loss of type‐II fibers in PROMM, their diminishing contribution to the overall carnosine signal may reduce the detectability of corresponding sub‐peaks. However, this interpretation remains speculative and warrants systematic validation. Future studies using dynamic MRS during standardized exercise are needed to clarify potential impairments of pH regulation in PROMM.

### 4.4 Limitations

This study has several limitations. First, it represents a pilot comparison in a relatively small cohort of PROMM patients and matched healthy volunteers. Strict inclusion criteria were necessary to ensure reliable carnosine quantification but further reduced the sample size, and the cross‐sectional design precludes conclusions on temporal dynamics. Nevertheless, the patients included here are under the clinical supervision of the Neuromuscular Centre Halle and are scheduled to receive follow‐up examinations every 3 years. This framework will enable longitudinal analyses of carnosine changes in combination with clinical parameters to probe its potential as a marker of disease progression in a future investigation.

Second, measurements were restricted to the quadriceps muscle, a proximal muscle with a relatively high proportion of type‐II fibers. This choice provides a strong pathophysiological rationale but limits generalizability to other muscle groups. Future studies should therefore also include muscles with a very low type‐II fiber content as internal reference tissues. Since carnosine alterations are expected to primarily reflect type‐II fiber degeneration, little or no differences between patients and healthy volunteers would be anticipated in such muscles, providing an important validation framework.

Third, carnosine intensities were normalized to the total creatine signal, which introduces two methodological constraints: the relatively low creatine amplitude increases sensitivity to quantification errors, and the large chemical shift difference between creatine and carnosine induces metabolite‐specific chemical‐shift displacements, potentially introducing additional variability in Carn/tCr ratios. Although referencing the unsuppressed water signal would in principle provide higher quantification accuracy and reduced chemical‐shift displacement, the available water reference acquisition in our protocol—performed without RF preparation for water suppression but with the same gradient scheme as the water‐suppressed scan—led to considerable variability in the resulting carnosine‐to‐water ratios. Therefore, total creatine was chosen as a more consistent internal reference. Future work should pursue absolute quantification of single metabolites to minimize these potential confounders. Moreover, localization sequences with broadband refocusing pulses, such as sLASER (semi‐Localized by Adiabatic SElective Refocusing) or STEAM (STimulated Echo Acquisition Mode), should be considered as alternatives to the PRESS technique to improve spatial accuracy [28]. Such advanced localization strategies may also facilitate more reliable separation of intra‐ and extramyocellular lipid resonances, which could enable IMCL quantification in future studies. Although IMCL may provide complementary metabolic insight into muscle fiber composition and lipid remodeling in PROMM, its reliable assessment was not feasible in the present dataset due to substantial overlap with EMCL and increased line broadening in fat‐infiltrated muscle.

## 5 Conclusion

This study demonstrates for the first time that localized ^1^H‐MRS can noninvasively detect reduced carnosine levels in proximal thigh muscles of patients with PROMM. This reduction most likely reflects the selective loss of type‐II fibers, a characteristic feature of the disease previously identified in histopathological studies. Our findings therefore suggest that carnosine may serve as an in vivo marker of fiber‐type‐specific degeneration in PROMM. Clinically, such a marker could be valuable for monitoring disease progression and guiding future therapeutic strategies. These results, however, should be regarded as preliminary. The present work was conducted as a pilot study in a small, cross‐sectional cohort and thus requires validation in larger populations. Future studies should also include muscles with different fiber‐type compositions and examine longitudinal changes in carnosine in conjunction with clinical parameters.

## Author Contributions

Conception: Alexander Gussew, Alexander Mensch, Dietrich Stoevesandt, Andreas Deistung. Methodology: Alexander Gussew, Maryam Kargaran, Maik Rothe. Conception, recruitment and clinical assessments: Maurits Gerhard Abraham Heuschen, Anna Katharina Kölsch, David Strube, Thomas Kendzierski. Data analysis and investigation: Alexander Gussew, Maryam Kargaran, Maik Rothe, Alexander Mensch. Writing – original draft preparation: Alexander Gussew; Writing – review and editing: Alexander Gussew, Maryam Kargaran, Maik Rothe, Walter A. Wohlgemuth, Andreas Deistung, Dietrich Stoevesandt, Alexander Mensch, Maurits Gerhard Abraham Heuschen, Anna Katharina Kölsch, David Strube, Thomas Kendzierski, Markus Otto. Funding acquisition: Walter A. Wohlgemuth, Alexander Gussew, Andreas Deistung, Markus Otto. Resources: Walter A. Wohlgemuth, Alexander Gussew, Andreas Deistung, Markus Otto, Alexander Mensch.

## Funding

This work was supported by Deutsche Forschungsgemeinschaft, GU 1108/4‐1, INST 271/406‐1 FUGG.

## Ethics Statement

The study was approved by the local ethics committee (protocol No.: 2022–012) and conducted in accordance with the Declaration of Helsinki.

## Consent

Written informed consent was obtained from all participants.

## Conflicts of Interest

The authors declare no conflicts of interest.

## Data Availability

The data supporting this study's findings are available from the corresponding author upon reasonable request.
